# Improved Pharmacokinetics of Aceclofenac Immediate Release Tablets Incorporating its Inclusion Complex with Hydroxypropyl-β-Cyclodextrin

**DOI:** 10.3797/scipharm.1509-07

**Published:** 2015-02-02

**Authors:** Sunita Dahiya, Atul Kaushik, Kamla Pathak

**Affiliations:** 1 Department of Pharmaceutics, Globus College of Pharmacy, Bangrasia, Bhopal-462045, MP, India; 2 Department of Pharmaceutics, IPS College of Pharmacy, Gwalior, MP, India; 3 Department of Pharmaceutics, Rajiv Academy for Pharmacy, Chhattikara, Mathura, UP, India

**Keywords:** Inclusion complex, Immediate release tablets, Bioavailability, *In vivo* studies, Pharmacokinetic parameters

## Abstract

The present investigation reports the various pharmacokinetic parameters of immediate release aceclofenac tablets incorporating its inclusion complex with hydroxypropyl-β-cyclodextrin. The tablets were prepared using aceclofenac: hydroxypropyl-β-cyclodextrin in a 1:1 molar ratio by the direct compression method (TKN). The results were compared with those of the marketed brand (MKT) and pure drug (TAC). The *P*-values indicated that mean plasma concentrations were significantly different among all three formulations administered (*P*<0.05, *P*<0.01). TKN showed significantly higher plasma levels when compared to the pure drug (*P*<0.01). The C_max_ and AUC_(0-∞)_ of TKN were significantly higher (*P*<0.05) compared to the pure drug and marketed formulation. Furthermore, the first-order overall elimination rate constant (K_el_) of TKN was also significantly higher (*P*<0.05) compared to the pure drug and its marketed formulation. These results suggested that tablets prepared by incorporating the AC-HPβCD inclusion complex (TKN) would provide a more rapid onset of pharmacological effects in comparison to the marketed formulation and pure drug.

## Introduction

Aceclofenac (AC), a phenyl acetic acid derivative, ({[2-(2,6-dichloroanilino)phenyl]acetyl}oxy)acetic acid, is a non-steroidal anti-inflammatory drug (NSAID) indicated for the symptomatic treatment of pain and inflammation with a reduced side effect profile, especially gastrointestinal events that are frequently experienced with NSAID therapy [1]. AC appears to be particularly well-tolerated among the NSAIDs, with a lower incidence of gastrointestinal adverse effects. This good tolerability profile results in a reduced withdrawal rate and hence, greater compliance with treatment. AC is a BCS class II drug (low solubility, high permeability) with a calculated log partition coefficient value of (log P) = 2.170; its poor aqueous solubility and wettability give rise to difficulties in pharmaceutical formulations for oral delivery which may lead to variable bioavailability. To overcome these drawbacks, increasing the aqueous solubility of AC is an important goal.

Cyclodextrins (CDs) are cyclic *α*-1,4-linked oligosaccharides of *α*-D-glucopyranose, containing a relatively hydrophobic central cavity and hydrophilic outer surface. CDs are able to form inclusion complexes with poorly water-soluble drugs. These inclusion complexes have been shown to improve the stability, solubility, dissolution rate, and bioavailability of the drugs [2]. The hydroxypropyl-*β*-cyclodextrin (HP*β*CD) is highly water-soluble due to both its chemical nature and amorphous property, and does not have limitations such as the renal toxicity associated with *β*-cyclodextrins (*β*CD) or other chemically modified cyclodextrins [3, 4].

The formulation and *in vitro* evaluation of the aceclofenac inclusion complex and its tablet formulation have been published earlier [5, 6] and described here briefly. The main objective of this work was to establish the potential of inclusion complexation as a bioavailability enhancement method for poorly water-soluble drugs. In this context, various pharmacokinetic parameters of the prepared tablet formulation were compared with that of the marketed brand and pure drug.

## Materials and Methods

Aceclofenac was generously provided by Kairav Chemicals, Ahmedabad. Hydroxypropyl-*β*-cyclodextrin (HPβCD) was purchased from Himedia Laboratories Pvt Ltd, Mumbai. All other chemicals used were of analytical or HPLC grade and doubly distilled water was used throughout the studies.

### Preparation of AC-HPβCD Inclusion Complex by the Kneading Method

For the kneaded product, 0.3541 g of AC and 1.3716 g HP*β*CD were mixed with 0.3 ml of water:ethanol (1:1) solvent for 20 min to produce dough, and the mixture was further kneaded in a mortar for 1 h to produce a paste of suitable consistency. The obtained semi-wet mass was dried in a vacuum desiccator at room temperature for 72 h using anhydrous calcium chloride as a desiccant and then passed through an 85-mesh sieve to obtain the kneaded product (KN).

### Preparation of Directly Compressible Tablets

Tablets were prepared incorporating either pure drug (AC) or its inclusion complex (KN) by the direct compression method using 10-mm punches on a hand-operated single punch tablet machine (Hicon, India). Each tablet formulation was coded using the prefix “T”. TKN consisted of an inclusion complex amount equivalent to 100 mg of AC, whereas TAC contained 100 mg of the pure drug AC. The detailed composition of tablets is given in Table 1.

**Tab. 1:**
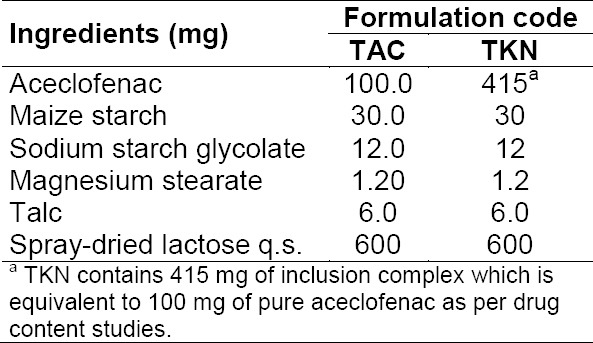
Composition of directly compressible tablets incorporating pure drug AC (TAC) and its inclusion complex (TKN)

### Evaluation of Tablets

Both diameter and thickness of the tablets were determined in millimeters using the average of three measurements in each case using Vernier calipers. Hardness was measured using the Monsanto hardness tester in terms of kg/sq.cm. The average hardness of ten tablets was taken to study the reproducibility. Ten tablets from each batch were exposed to the friability test apparatus for 100 rotations and the percentage loss in weight was measured against the initial weight. Further, 20 tablets were selected at random from each formulated batch to check the uniformity of weight using an electronic balance. The average weight and maximum percent deviation (positive and negative) were determined. The disintegration test was carried out using a disintegration test apparatus USP using distilled water as the disintegration medium. One tablet was introduced into each tube and a disc was added to each tube. Assembly was suspended in the beaker containing 900 ml distilled water. The time for disintegration of all six tablets was noted. For the assay, 20 tablets were selected at random from each batch of formulated tablets and powdered. Ten mg powder from each tablet batch was diluted to 10 ml using methanol and the resultant solution was shaken for one min using a vortex mixer. All the samples were filtered using Whatman No.1 filter paper. From this, 0.5 ml solution was withdrawn and diluted to 10 ml with methanol. The concentrations of AC in the filtrates were determined spectrophotometrically at 275 nm with reference to a suitably constructed calibration curve of AC in methanol.

### In Vitro Dissolution Tests of Tablets

*In vitro* dissolution studies for the formulated tablets were carried out using a USP 23 type II paddle dissolution apparatus at 37°C temperature and 100 rpm stirring rate, using 900 ml phosphate buffer pH 7.5 as a dissolution medium (n=3). Samples were withdrawn at predetermined time intervals and replaced with fresh dissolution medium, suitably diluted, and analyzed at 275 nm.

### Comparison with Marketed Brands

The prepared tablet formulation was compared with popular immediate release marketed tablets consisting of 100 mg of AC and coded as MKT.

### Pharmacokinetic Study of the Tablet Formulation Incorporating the Inclusion Complex (TKN), Marketed Tablet (MKT), and Pure Drug (TAC)

### Protocol for Pharmacokinetic Studies in Animals

The pharmacokinetic studies were carried out on Wistar rats. Male Wistar rats (weighing 200–250 g) were obtained from the breeding centre of Rajiv Academy for Pharmacy, Mathura. They were housed in elevated wire cages, three animals per cage, with free access to food (Lipton Feed, Mumbai, India) and water. The pre-clinical study protocol was approved by the Institutional Animal Ethical Committee, Rajiv Academy for Pharmacy, Mathura (Approval No: IAEC/12/09).

### Pharmacokinetic Studies

The overnight-fasted rats were divided into three groups (n=3) and treated as follows:

Group I: Pure aceclofenac (10 mg/kg) in 0.5% CMC; p.o.

Group II: Powdered marketed aceclofenac tablet formulation (10 mg/kg) in 0.5% CMC; p.o.

Group III: Powdered formulated tablet containing aceclofenac inclusion complex (10 mg/kg) in 0.5% CMC; p.o.

Then, blood samples (0.2 ml) were withdrawn through the jugular vein at 0.17, 0.33, 0.5, 0.67, 1, 2, 4, 6, and 8 h of post-dose, into heparinized tubes. The plasma was separated immediately using cold centrifugation (Remi Equipments Ltd., Mumbai) at 2500 rpm for 20 min. The collected plasma (100 µl) samples were stored at −20°C until analysis.

### Chromatographic Conditions

A sensitive high-performance liquid chromatographic (HPLC) method was used to analyze aceclofenac in plasma. The HPLC system (Shimadzu Class VP series having Class VP 6.12 version software) with two pumps (LC-10AT VP), a variable wavelength programmable UV/VIS detector (SPD-10A VP), a system controller (SCL-10A VP), and an RP C-18 column (Hypersil BDS C18; 250 cm × 4.6 mm; 5 µ) was used. The mobile phase was methanol + 0.3% TEA pH 5.0 (60:40 v/v) and the flow rate was 1.0 ml/min. The detection wavelength was 275 nm.

### Preparation of the Calibration Curve in Plasma

Ten mg of the drug was dissolved in 10 ml of plasma (spiked with 1 ml of drug-free methanol and vortexed) to get a concentration of 1000 µg/ml. From this, 1 ml was diluted to 10 ml (100 µg/ml) and again, from this, 1 ml was diluted to 10 ml (10 µg/ml). This solution was suitably diluted to prepare working standard solutions containing 100–5000 ng/ml of aceclofenac.

### Preparation of Sample Solutions

To 100 µl of plasma, 200 µl of diluent was added and mixed for a minute. To this, further, diluent was added (700 µl) up to 1 ml. The resulting solution was vortexed for 60 s and centrifuged at 3000 rpm for 10 min. The supernatant layer was separated and analyzed using the HPLC system. The concentrations of the aceclofenac present in the plasma samples were calculated using the calibration curve. The blank plasma samples were analyzed prior to the analysis of aceclofenac standard preparations.

### Validation of the HPLC Method

The method was validated with respect to accuracy, precision, level of detection, and level of quantification. The limit of quantification for AC in plasma was also reported. The relationship between the concentration and peak area was found within the range 0.1–5 µg/ml during linearity studies. Quality control points at low, medium, and high levels were used to determine absolute recovery and within-day (intraday) and between-day (interday) precision and accuracy [7].

### Pharmacokinetic and Statistical Analysis

The pharmacokinetic parameters were directly determined or calculated by the standard compartmental model of independent analysis. Both the maximum plasma concentration (C_max_) and time to peak plasma concentration (t_max_) were obtained directly from the data. The elimination half-life (t_1/2_) was calculated as 0.693/K_el_ where K_el_ is the apparent elimination rate constant. K_el_ was in turn calculated as the slope of the linear regression line of natural log-transformed plasma concentrations. The last five to six quantifiable levels were used to determine K_el._ The absorption rate constant (K_a_) and lag time were derived using method of residuals, whereas absorption half-life (t_1/2ab_) was obtained as 0.693/K_a._ The area under the plasma concentration-time curve (AUC_0-t*_) was calculated from the measured levels, from time zero to the last quantifiable level, by the linear trapezoidal rule. AUC_0-∞_ was calculated according to the following formula:

AUC_0-∞_ = AUC_0-t*_ + C*/K_el_

where C* is the last quantifiable plasma level

The area under the first moment curve (AUMC) was obtained from a plot of the product of plasma drug concentration and time *vs*. time t from zero to infinity using the trapezoidal rule. Mean resident time (MRT) is defined as the average amount of time spent by the drug in the body before being eliminated, and was obtained as the AUMC/AUC. Parametric statistical evaluation of the data was performed by Student’s t-test and one-way variance analysis followed by Tukey multiple comparisons employing GraphPad InStat 8.0 for Windows.

## Results and Discussion

### Evaluation of Tablets and Comparison with Marketed Brands

All tablet formulations were found satisfactory with respect to physical parameters and compendial standards. Moreover, the DP_10_ (percentage drug released at 10 min) values for TAC, MKT, and TKN were found to be 6.39, 52.82, and 100.69, respectively.

### Pharmacokinetics of Tablet Formulations Incorporating the Inclusion Complex (TKN), Marketed Tablet (MKT), and Pure Drug (TAC)

### HPLC Method

**Fig. 1:**
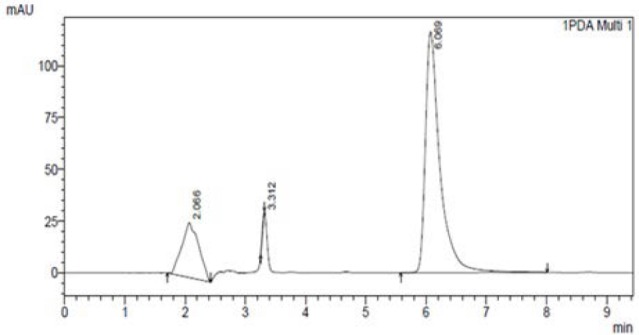
HPLC chromatogram of AC in plasma

**Fig. 2:**
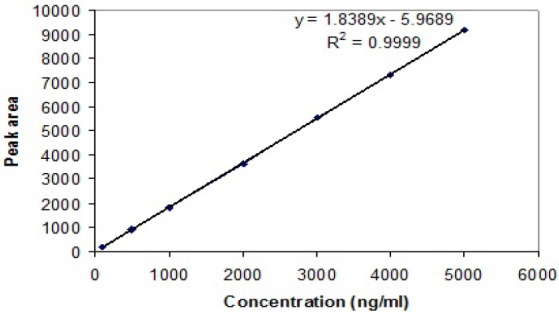
Calibration and linearity data

The described analytical method used for the measurement of AC was found to be accurate and sensitive and in the HPLC method, no interference was observed in rat plasma. The limit of quantification for AC in plasma was 0.1 µg/ml. The level of detection was 1 ng/ml and the level of quantification was 5 ng/ml. The HPLC elution spectrum for AC was well-resolved and the retention time for AC was 6.069 min as depicted in Figure 1. Table 2 summarizes the within- and between-day precision and accuracy. Quality control points at low, medium, and high levels (150, 450, and 900 ng/ml) were used to determine the absolute recovery and within- and between-day precision and accuracy. The linearity achieved for this assay (0.1 to 5 µg/ml) effectively covered the therapeutic range, and the linearity was assessed from the graph of peak height versus concentration of AC. The calibration curve of AC constructed in the range of 0.1–5.0 µg/ml was found linear over this range with the r^2^ value of 0.9999 (n=6) as shown in Figure 2. An internal standard was not used in the study because the high recovery of extraction with the proposed method allowed the non-use of internal standard without compromising precision and accuracy [8].

**Tab. 2:**
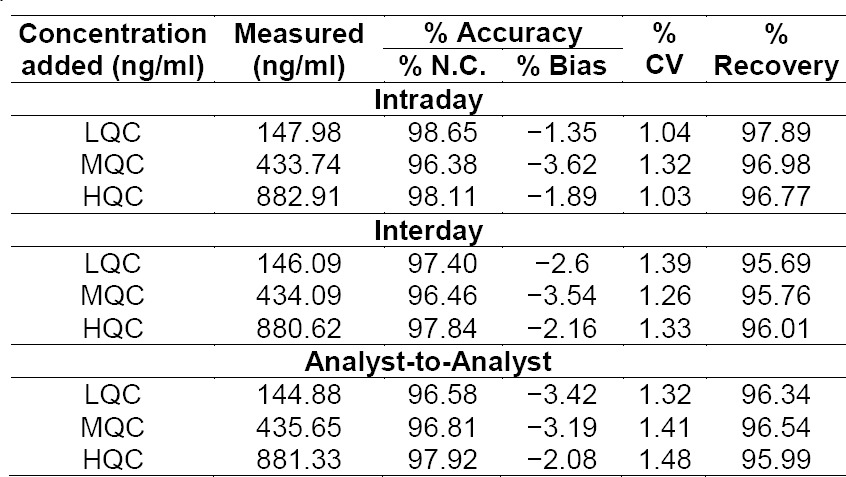
Accuracy and precision of the HPLC method for the determination of AC in plasma

### Pharmacokinetic Analysis

**Fig. 3:**
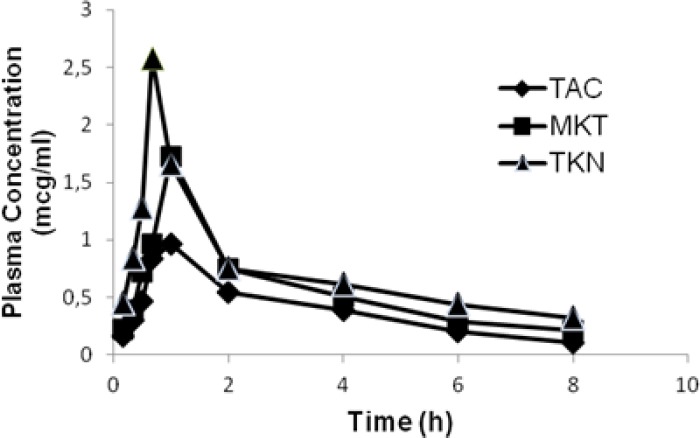
Comparative plasma concentration vs. time profiles of TAC, MKT, and TKN

The comparative plasma concentration vs. time profiles are depicted in Figure 3. The calculated pharmacokinetic parameters are presented in Table 3. Aceclofenac absorption after oral administration was rapid with all three groups as indicated by t_max_ values. However, the C_max_ value was significantly higher with TKN (C_max_ 2.58 µg/ml) compared to the pure drug (C_max_=0.96 µg/ml) and marketed tablet (C_max_=1.65 µg/ml) (*P*<0.05), indicating a high absorption rate of the drug from the tablet incorporating the inclusion complex as compared to that of the marketed tablet as well as the pure drug. Following the administration of tablets, the mean area under the plasma concentration–time curve (AUC) was found to be 6.237±0.550 µg.h/ml, 3.571±0.319 µg.h/ml, and 5.061±0.154 µg.h/ml for the tablet containing the inclusion complex (TKN), pure AC (TAC), and the marketed tablet (MKT), respectively. Thus, the results revealed that there was about 1.75- and 1.2-fold enhancements in the extent of absorption of AC from TKN as compared to the pure drug and marketed tablet, respectively. Moreover, maximum plasma concentration appeared at a shorter time after dosing with TKN (t_max_= 0.67 h) than with pure AC (t_max_ = 1 h) and the marketed tablet (t_max_ = 1 h).

**Tab. 3:**
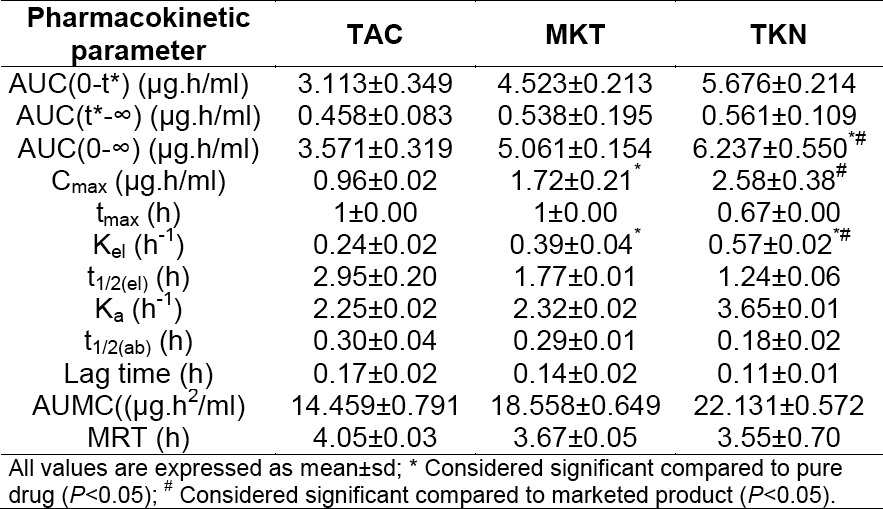
Comparative pharmacokinetic parameters of TAC, MKT, and TKN

The elimination half-life (t_1/2(el)_) of AC with TKN was less indicating that the drug was getting eliminated from the body rapidly, which in turn was supported by less mean residence time (MRT) and a high elimination rate constant value (K_el_) of TKN in comparison with the marketed formulation and pure drug. The t_1/2(el)_, MRT, and K_el_ values indicated that the pure drug remained in the body for more time when compared with the marketed and TKN formulations. TKN showed a high AUC value indicating the greater extent of drug absorption from the inclusion complex. Thus, low t_max_, high C_max_, and high AUC values together indicated the improved bioavailability and rapid absorption of AC from the inclusion complex in comparison with the marketed formulation and pure drug. This could be due to improved solubility and dissolution rate of the drug from the inclusion complex.

### Statistical Analysis

In all cases, statistical significance was determined at the 95% confidence level. The statistical parameters of plasma concentrations-time profiles of formulations are summarized in Table 4. The *P*-values indicated that mean plasma concentrations were significantly different among all three formulations administered (*P*<0.05, *P*<0.01). TKN showed very significantly higher plasma levels when compared to the pure drug (*P*<0.01). The C_max_ and AUC_(0-∞)_ of TKN were significantly higher (*P*<0.05) compared to the pure drug and marketed formulation. Furthermore, the K_el_ of TKN was also significantly higher (*P*<0.05) compared to the pure drug and its marketed formulation. These results suggested that TKN would have been provided a more rapid onset of its pharmacological effect in comparison to the marketed formulation and pure drug, and had been rapidly eliminated from the body when compared with marketed formulation and TKN.

**Tab. 4:**
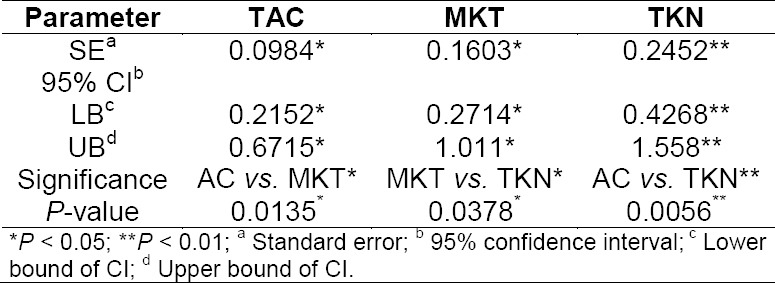
Summary of statistical parameters of plasma concentration-time profiles of formulations

## Conclusion

The immediate release AC tablet formulation incorporating its inclusion complex with HPβCD produced a shorter t_max_ and a higher C_max_ as well as a much better extent of absorption as compared to the tablet containing the pure drug and its marketed formulation. The pharmacokinetic study also indicated rapid absorption and higher bioavailability of the drug from the prepared tablets in comparison to the marketed formulation as evidenced by higher AUC and C_max_, and lower t_max_ values. The potential results obtained through these studies suggested that the inclusion complex represents a valuable approach for development of a better oral dosage form as compared to that existing in the commercial market and if scaled-up, may be promising for the formulation development of other poorly water-soluble drugs from the viewpoint of industry.
